# Super-resolution microscopy and deep learning methods: what can they bring to neuroscience: from neuron to 3D spine segmentation

**DOI:** 10.3389/fninf.2025.1630133

**Published:** 2025-09-29

**Authors:** Paul Nazac, Shengyan Xu, Victor Breton, David Boulet, Lydia Danglot

**Affiliations:** ^1^Institute of Psychiatry and Neuroscience of Paris (IPNP), Université Paris Cité, INSERM U1266, Membrane Traffic in Healthy and Diseased Brain Team, Paris, France; ^2^Membrane Mechanics and Dynamics of Intracellular Signaling Laboratory, Institut Curie Research Center, CNRS UMR3666, INSERM U1339, PSL Research University, Paris, France

**Keywords:** super-resolution (SR), deep learning, neuron, dendrite, dendritic spine, labeling, segmentation (image processing), probe

## Abstract

In recent years, advances in microscopy and the development of novel fluorescent probes have significantly improved neuronal imaging. Many neuropsychiatric disorders are characterized by alterations in neuronal arborization, neuronal loss—as seen in Parkinson’s disease—or synaptic loss, as in Alzheimer’s disease. Neurodevelopmental disorders can also impact dendritic spine morphogenesis, as observed in autism spectrum disorders and schizophrenia. In this review, we provide an overview of the various labeling and microscopy techniques available to visualize neuronal structure, including dendritic spines and synapses. Particular attention is given to available fluorescent probes, recent technological advances in super-resolution microscopy (SIM, STED, STORM, MINFLUX), and segmentation methods. Aimed at biologists, this review presents both classical segmentation approaches and recent tools based on deep learning methods, with the goal of remaining accessible to readers without programming expertise.

## Neuronal morphology and synaptopathies

**Figure 1:**
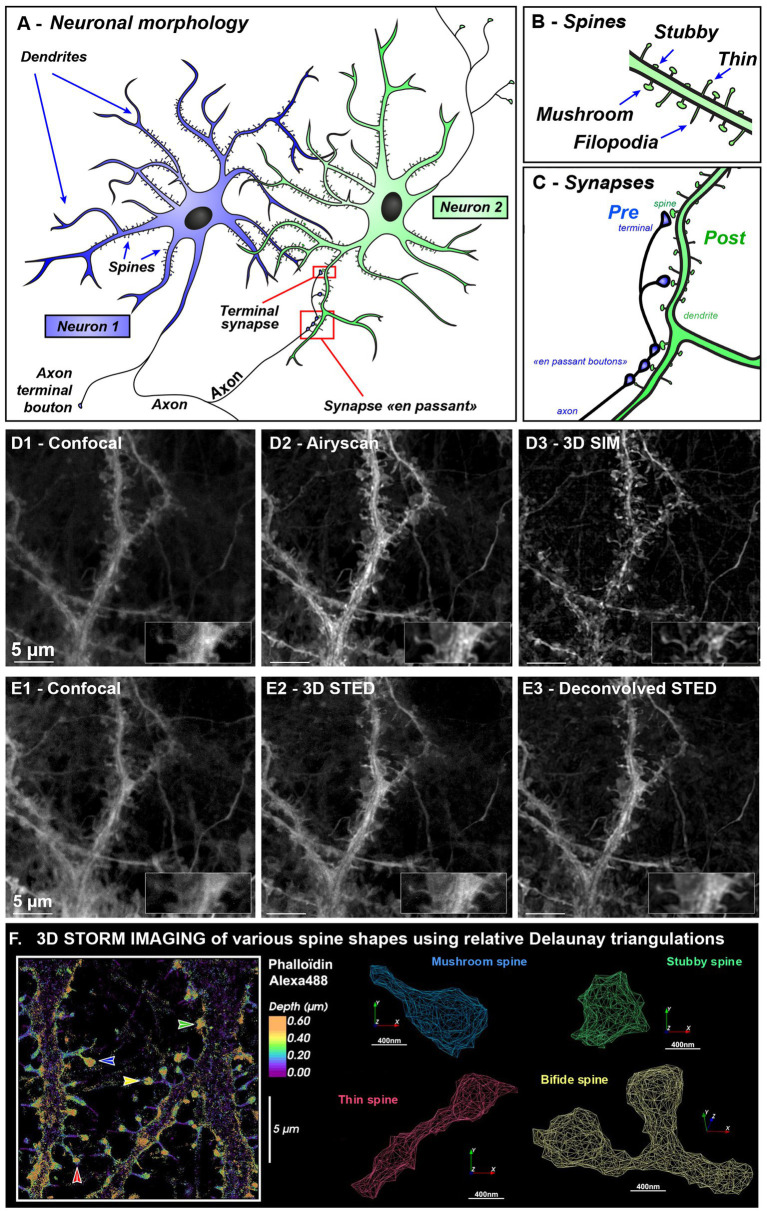
Neuronal and spine morphologies revealed using various microscopy modalities illustrating resolution gain needed for spine imaging. **(A–C)** Morphology of communication between two neurons **(A)**. A neuron is made up of a cell body, an axon, and several dendrites. The axon can divide in collaterals branches that can contact several other neurons and release neurotransmitter at synaptic sites. Synapses are dedicated contact sites between soma or dendrites that receives information from neighboring axons. Synapse can be present at the very end of the axon (at the presynaptic terminal) or can establish “contact en passant” along the axon. On the dendrite, the synaptic zone is called post-synaptic. Blue Neuron is sending information via his axon and green neuron is receiving information on different dendritic spines **(B)** leading to the formation of synapses **(C)**. **(D,E)** Correlative images of hippocampal neurons after 21 days *in vitro* labeled with MemBright Cy3.5 probe **(D1–D3)** were acquired on Zeiss Elyra PS1 equipped with Airyscan and SIM allowing correlative images of various microscopy modalities while **(E1–E3)** were imaged on Leica confocal STED 3DX. The same sample and region was used for both **(D,E)**. The inset display a magnified portion of the dendrite harboring one thin (left) and mushroom spine (right). Deconvolution was done using Classic Maximum Likelihood Estimation in Huygens software. Scale bar: 5 μm. **(F)** 3D STORM imaging of dendritic spines class was done using Alexa488-Phalloidin that labels spine heads and neck. All STORM localizations, were pooled and external envelope was reconstructed using Delauney triangulation. In contrast to membrane labeling, here spine head is underestimated since actin is not filling all spine head volume especially close to the PSD. Various spine types are shown with colored arrows. Stubby spine (with no neck) is in green, mushroom spine (with short neck and big head) is in blue, thin spine (with long neck and small head) is in red, and a bifid spine is in yellow.

Neuronal cells are communicating through billions of synapses. Axons can contact dendrite directly (shaft synapse) or established contact with a dendritic membrane protrusion called dendritic spine (Bucher et al., 2020) (Figure 1A). Dendritic spines are highly dynamic structures, full of actin, changing their shape and numbers during development, ageing and learning (Rao and Craig, 2000; Zhang and Benson, 2000; Konietzny et al., 2017; Bucher et al., 2020). They are filamentous during development [called filopodia (Herms and Dorostkar, 2016), Figure 1B] and can then either retract to form stubby-type spines (without neck) (Harris, 1999) or develop a head (Hotulainen and Hoogenraad, 2010; Kashiwagi and Okabe, 2021) during or after learning. Spine with heads can be categorized into “Thin” (long spine with small heads) or “mushroom” (short spine with larger head and restricted neck) (Harris, 2020). Such changes affect synaptic function and plasticity at the cellular level. For the past decades, the importance of spines shape in neuropathological disorder-related disease has emerged with the development of biochemical, imaging and analysis tools. In particular, changes in dendritic spine shape and number is associated with a large number of brain disorders that involve deficits in information processing and cognition (Forrest et al., 2018). Recent evidence supports altered synaptic connectivity and plasticity within developmental stages in children and adolescents (ASD, Autism Spectrum Disorders and schizophrenia) but also in ageing associated disease (Alzheimer’s disease with memory deficit) (Glantz and Lewis, 2000; Selkoe, 2002; Tackenberg et al., 2009; Hutsler and Zhang, 2010). Specifically, accumulating neuropathological evidences points toward synapse and dendritic spine loss in schizophrenia (Garey et al., 1998) and Alzheimer’s disease (Boros et al., 2017; Forrest et al., 2018; Rao et al., 2022), whereas ASD displays an increased spine number and immature spine shape (Hutsler and Zhang, 2010). Hence dendritic spines could be seen as a common substrate to study neuropsychiatric disorders involving cognitive deficits.

## Imaging neurons and dendritic spines with cytosolic and membrane probes

Neuronal morphology has traditionally been visualized using confocal microscopy after transfection of cytosolic GFP, multicolor brainbow variants (Livet et al., 2007) or biolistic delivery of DiIC₁₈, a lipophilic dye diffusing in the entire membrane (see Okabe, 2020; Wouterlood, 2023 for reviews). DID which is an oil version of DiIC18 has also been used on hippocampal neurons in culture. However, variability in intensity levels between cells, complicated quantitative segmentation (Collot et al., 2019). Spine morphogenesis could be monitored using spinning-disk live imaging with actin-GFP reporters (Supplementary Figure S1A). On fixed samples, spines are efficiently labeled with fluorescent phalloidin, a toxin that binds specifically to F-actin (Supplementary Figures S1B–D). When using phalloidin or cytosolic GFP, dendritic spine necks (red arrows) typically exhibit lower fluorescence compared to spine heads (green arrow), making them more difficult to visualize and quantify. The use of new variant of membranous GFP [Addgene 117,858 (Wu et al., 2018); Supplementary Figures S1E,F] improved neck detection. Live Membrane can also be stained indirectly using fluorescent Wheat Germ Agglutinin that are lectins binding to carbohydrates present at the cell surface. However, this labelling is dependent on carbohydrate distribution which can be sometimes expressed in a punctate pattern complexifying neuronal segmentation (Collot et al., 2019). More recently, we developed the MemBright™ probes (Collot et al., 2019) that are lipophilic fluorescent dyes labeling any plasma membrane after 5 min incubation in cell culture media (Supplementary Figures S1B–D in red). MemBright™ offers the key advantage of labeling all cell types without requiring transfection, making it suitable for both live and fixed samples. By uniformly integrating into the plasma membrane, it enables clear visualization of both spine necks and heads (Supplementary Figures S1B–D, red arrows and Supplementary Figures S1G–I), facilitating accurate neuronal segmentation.

The advent of super-resolution microscopy has revolutionized neuroimaging by enabling 3D-visualization of nanoscopic nervous system components. Techniques such as **Structured Illumination Microscopy (SIM)** and **Airyscan technology** now routinely achieve resolutions of approximately 100 to 140 nm, respectively (see Figures 1D2,D3 and Glossary for detailed optical principle and comparison of super-resolution techniques with resolution and applications). These enhanced resolutions, combined with improved signal-to-noise ratios, enable the visualization of small structures—such as pre- or post-synaptic protein clusters and small organelles like endosomes. Such fine structures can then be assessed for their synaptic localization using **Ripley’s Function**. We developed **Icy SODA plugin** (Lagache et al., 2018) to detect coupling between non overlapping pre and post synaptic proteins and accurately measure their coupling distances. SIM microscopy, with its wide field of view, enables large-scale screening of synaptic structures. From just 15 SIM images, we were able to analyze over 45,000 synapsin clusters and determine their associations with post-synaptic markers such as PSD95 (mean distance: 107 ± 73 nm) and Homer (138 ± 89 nm). The combination of SIM with ICY SODA plugin thus provides a powerful method for molecular mapping of synapse, facilitating the rapid identification of potential synaptopathies (Breton et al., 2024; De Koninck et al., 2024).

For live-cell imaging, Airyscan’s inline acquisition technology provides image quality far superior to that of spinning disks and operates 4 to 5 times faster than conventional confocal microscopy, enabling dynamic monitoring of nanoscale synaptic components over time. Airyscan can provide isotropic pixels paving the way to improved 3D spine morphology reconstruction (Figure 1D2 and Supplementary Figures S1B–I).

**3D-STED** microscopy (Figures 1E2,E3 & Glossary) is particularly well-suited for tissue imaging, enabling resolution of synaptic contacts even in depth. Unlike SIM, it is less prone to reconstruction ringing-artifacts surrounding clusters. The primary limitation in tissue imaging, lies in the scattering nature of neural tissue, which restricts the depth of light penetration. This constraint can be overcome by implementing a tissue clearing step, which facilitates light propagation in thick samples (ranging from 0.5 to 1 mm in thickness). We recently employed such a combined approach (Cauzzo et al., 2024), clearing thick brain sections from transgenic mice expressing cytosolic GFP in Purkinje cells. This enabled us to perform correlative imaging across scales—from low-magnification mosaic imaging (20x, covering several millimeters in width) to high-magnification 3D-STED imaging (93x). This multiscale strategy allowed us to correlate cellular population organization, neuronal dendritic arborization, and the morphology of dendritic spines, including necks as narrow as a 100 nm.

Single-molecule localization microscopy (SMLM), with localization precisions of 10–30 nm for **STORM** and as low as 2–3 nm for MINFLUX, provides nanoscopic resolution previously reserved to electron microscopy. It enabled detailed visualization of actin–spectrin networks around clathrin-coated pits (Bingham et al., 2023; Wernert et al., 2024) and dendritic spines (Breton et al., 2024; Saladin et al., 2024). 3D-STORM imaging using MemBright or fluorescent phalloidin, combined with Delaunay triangulation (see Glossary), can reveal spine neck constrictions characteristic of mushroom and thin spines (Figure 1F). Recently, MINFLUX has even tracked dynein stepping in live neurons (Schleske et al., 2024).

## Neuronal and dendritic spine segmentation pipeline

Much emphasis has been put in the past decades on the correlation between structural changes (termed as synaptic plasticity) and neurodegenerative diseases. Dendritic complexity can be evaluated with the total dendritic length along with the number and distribution of their branching points. Pyramidal cell contains roughly 30,000 synapses and dendritic total length is 3 times longer in human cortex (14.5 mm) than in macaque (6.2 mm) or mice (5.3 mm)(Mohan et al., 2015). Pyramidal cells in the human prefrontal cortex have 72% more dendritic spines than macaques and four times more than squirrel monkeys or the mouse motor cortex (DeFelipe, 2011). Spine density can range from 1–4 spine/μm in rat and mice hippocampal neurons (Papa et al., 1995; Nwabuisi-Heath et al., 2012) up to 15/μm in cerebellum (Napper and Harvey, 1988). Thus, precise, high-throughput quantification of spine morphology is critical.

Spine segmentation pipelines generally fell into two categories: rule-based or data-driven approaches. Computational methods that can segment and quantitate dendritic spines in either 2D or 3D imaging have been exhaustively reviewed in 2020 (Okabe, 2020), we will try here to complete this view with the last recent approaches.

Rule-based pipelines rely on features established by image analysts (or neurobiologists) regarding dendritic spines characteristics. Most segmentation pipelines adopted a two-step strategy: first segmenting the entire dendrite tree and then extracting the spines.

Dendrite segmentation can rely on intensity thresholding, such as global Otsu (Li et al., 2022), multilevel thresholding (Kashiwagi et al., 2019) or adaptive thresholding (Ekaterina et al., 2023). Platforms like Vaa3D offer diverse segmentation methods with user-friendly visualization interface (Peng et al., 2010).

Uniform staining quality significantly enhanced the performance of threshold-based approaches. When image quality is insufficient for reliable segmentation, preprocessing steps such as smoothing or denoising can be required (Smirnov et al., 2018; Das et al., 2021; Argunşah et al., 2022). Targeting the inherent signal heterogeneity in confocal and two-photon images, the SmRG algorithm integrated Region Growing (RG) procedure with a mixture model describing the signal statistics (Callara et al., 2020). This allows SmRG to calculate local thresholds for iteratively growing segmented structure, thus enabling the 3D segmentation of complex neurons.

## Dendritic spine segmentation strategies

**Figure 2:**
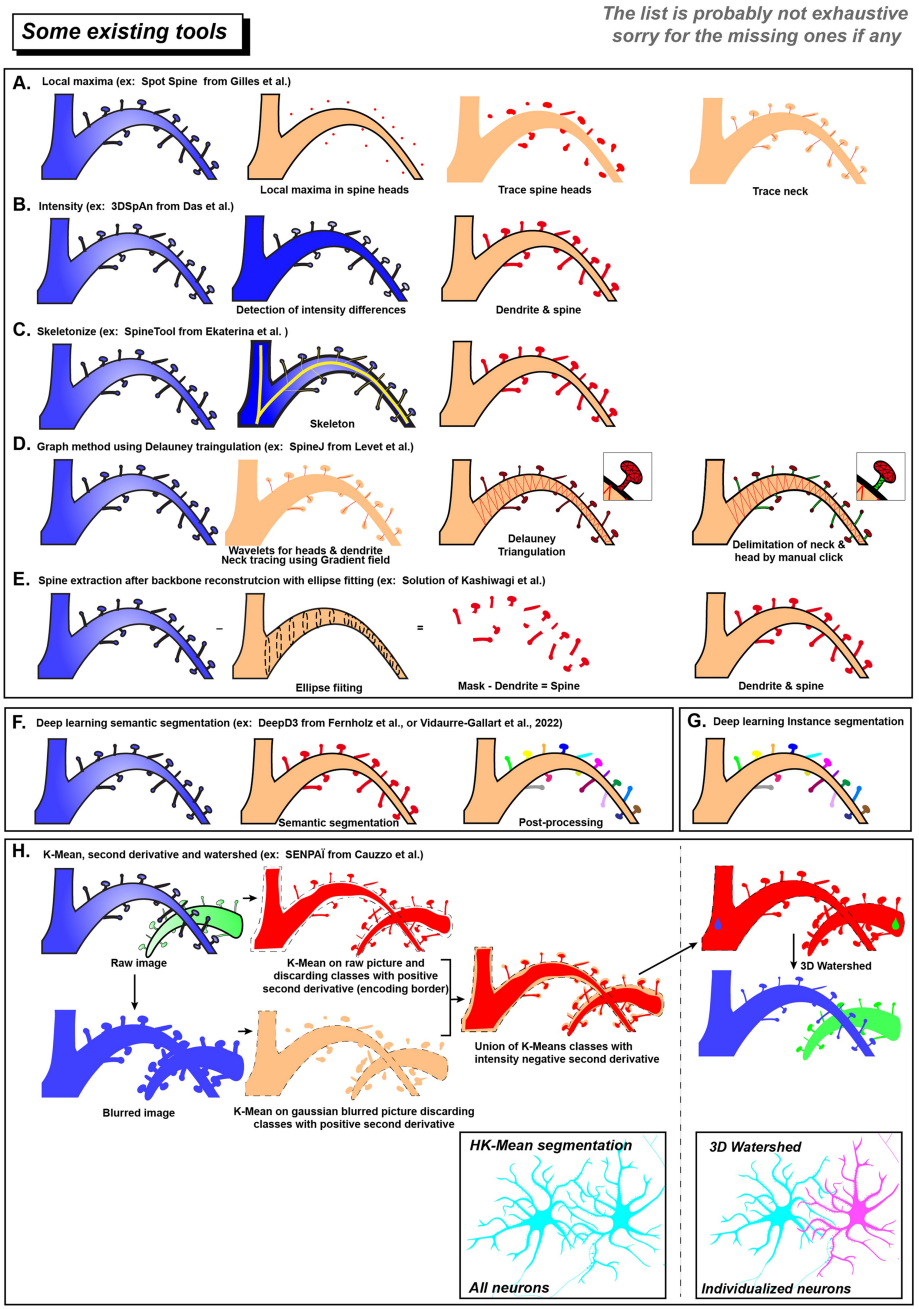
List of existing tool for spines and neurons segmentation. Schematic overview of dendritic spines segmentations using different ruled based approaches **(A–E)** or data driven modalities **(F–H)**. Deep learning methods can use semantic segmentation **(F)** or instance segmentation **(G)**. **(H)** SENPAÏ Tool for neuron and spine segmentation is using both ruled based and data driven approaches. In the end, neurons in a dense population will be segmented as an individual cell (blue and magenta).

One strategy uses the differences in pixel intensity between dendritic spines and shaft after staining (see Figures 2A,B). The implementation of intensity-based criteria varies among segmentation approaches. For example, the Spot Spine assumes spine heads have stronger signals, locating and segmenting them by finding local intensity maxima (Gilles et al., 2024). In contrast, 3dSpAn can handle cases where spine are weaker (light blue in Figure 2B) than the shaft (Figure 2B, dark blue). It iteratively isolates spine structures by applying multi-scale morphological opening operations (Das et al., 2021).

Skeletonization is another strategy for spine segmentation (Figure 2C). By using the fact that spines are considerably shorter than dendritic shaft, Rusakov et al. and SpineTool skeletonized the dendritic structure and identified short branches as potential spines (Rusakov and Stewart, 1995; Ekaterina et al., 2023). In contrast, SpineJ (Figure 2D) infers a graph structure from the generated skeleton (Levet et al., 2020). Then it identifies spine by analyzing nodes topological properties (e.g., leaf nodes and their connectivity patterns).

Other strategies first reconstructed the dendritic shaft and then considered external protrusions as spine candidates. Kashiwagi et al. (2019) proposed to fit the dendritic shaft using elliptical cross-sections (Figure 2E). Protrusions extending beyond the reconstructed shaft are regarded as candidate spines and are subsequently filtered according to geometric characteristics (volume or axial elongation).

A key advantage of rule-based methods is their strong interpretability, without the need for annotation, making them ideal when annotated datasets are scarce. However, since these methods rely on fixed logic, ensuring consistency in imaging conditions (microscopy modality, laser intensity, magnification and numerical aperture…) is crucial for optimal performance.

Data-driven pipelines, which include traditional machine learning methods [e.g., clustering methods like **K-means** (MacQueen, 1967; Jain et al., 1999)] see Glossary and deep learning methods [like the widely used **U-Net** for semantic segmentation (Ronneberger et al., 2015)], have been effectively applied in dendritic spines analysis.

Xiao et al. trained a fully convolutional network (**FCN**, see Glossary) to achieve automated detection of dendritic spines in 2D images (Xiao et al., 2018). Regarding 3D dendritic spine segmentation, Vidaurre-Gallart et al. elegantly developed a dataset of *in vitro* confocal images from healthy human tissue and trained a 3D U-Net model for segmentation (Vidaurre-Gallart et al., 2022). They created an image user interface allowing post-processing, such as reconnecting isolated dendrites and applying the watershed algorithm to resolve overlapping dendritic spines.

Some other tools like DeepD3 offers capabilities for both training and prediction and provides dendritic spine and dendrite segmentation (Fernholz et al., 2024). The deep learning framework utilizes a diverse dataset derived from two-photon and confocal imaging techniques, incorporating both *in vivo* and *in vitro* data, and supporting various fluorophores (tdTomato, Alexa-594, EGFP). DeepD3 employs an enhanced **U-Net** architecture with a **dual-decoder network**, generating separate segmentation masks for dendrites and spines. Post-processing includes filtering small segments, dilating dendritic maps to ensure that spine candidates are close to dendrites, and applying methods like flood-filling (or threshold-based 3D connected component analysis) for region of interest detection, with adjustable parameters to refine segmentation results. A graphical user interface is provided for both training and prediction.

In addition to develop new segmentation models, the extension of existing deep learning tools plays an important role in enhancing the accessibility of these technologies for neuroscientists. As an example, Schünemann et al. (2025) employed the Zeiss arivis Cloud platform to train and deploy a model for human dendritic spine morphology analysis. Similarly, the recent RESPAN pipeline (Garcia et al., 2024) integrates CSBDeep (Weigert et al., 2018) for image restoration (improving signal and contrast) with nnU-Net (Isensee et al., 2021) for spine segmentation, offering a combined workflow solution.

Other data-driven techniques such as clustering algorithms, operating without manual annotation, are also crucial. These approaches leverage intrinsic data properties to discern structures, offering distinct advantages for complex biological datasets where extensive annotation is prohibitive notably in nanoscale imaging.

Some techniques such as the multiphoton confocal microscopy allow the visualization of very dense neurons arborization. The resolution/**SNR** are sufficient to resolve spine head but mitigate spine neck detection. Previously, we developed some algorithm to infer neck central line using geodesic distances (Jain et al., 2021). However, since neck width was proposed to be related to electrical resistance, investing much effort in super-resolution technique would make neck detection more accurate and biologically relevant. The use of the 3D-STED microscopy, overcoming the diffraction of light, enables the characterization of these small protrusions. Segmenting these neurons requires the use of algorithms but only a few of them are able to deal with a complex cell packing (Li et al., 2019; Milligan et al., 2019; Callara et al., 2020).

We recently develop a new framework called SENPAI (SEgmentation of Neurons using PArtial derivative Information) that extract information of single neurons at micro and nanoscale within brain tissue. As a proof of concept, we considered L7-GFP transgenic mice that express cytosolic GFP within cerebellar Purkinje cells. SENPAI is working with a two-step segmentation and parcellation process which gives back the morphology at the nanoscale when combined with super-resolution.

SENPAI constitute a topological informed data driven approach to neuronal reconstruction. We used k-means clustering (Figure 2H) on raw or 3D Gaussian smoothed image to retrieve, respectively, tiny details (spine neck) or higher structures (dendrites & spine heads). To decipher border of the object (neuron or spine), we used a k-means scalable algorithm exploiting spatial derivatives of intensity. Indeed, the fluorescence is smoothly decreased at the neuronal border. We took advantage of intensity second derivatives that are negative within the inner edge of this intensity transition. Classes with positive second derivatives were considered as background whereas neuronal shape of interest is found in highest average intensity classes that present negative second derivatives in the 3 directions (Figure 2H).

The second step of SENPAI is the parcellation of the segmented image which exploits topographic distances. To separate neurons from the entire neuronal population, SENPAI uses the 3D watershed transform relying on cell body seeds used as neuronal core. This separation is applied on the previous segmentation mask, making the connection between dendritic branch and the related cellular body seed colorizing them into a single neuronal entity. In high-resolution datasets when spine necks are not detectable, the same strategy is used to assign small spines to a specific dendritic branch, which serves as the neuronal core. Thus, our SENPAI pipeline can isolate and extract neuron’s morphology (Figure 2H, pink neuron) from neuronal branches to dendritic spines even in a dense arborization context like in brain tissue.

## Discussion

### Enhancing segmentation performance: architectural and non-architectural strategies

Both rule-based and data-driven methods have made significant progress in 3D dendritic spine segmentation, each offering unique advantages in terms of interpretability and robustness.

Data-driven approaches, especially deep learning, enable different levels of segmentation. **Semantic segmentation** (Figure 2F & Glossary) classifies all spine pixels into a single category (spine) (Long et al., 2015); **instance segmentation** (Figure 2G) further identifies and distinguishes each individual spine instance (He et al., 2017); and **panoptic segmentation** integrates both, distinguishing individual spine instances while assigning a semantic label to every pixel, including the dendritic shaft, background, and other cellular structures (Kirillov et al., 2019).

Moving forward, combining the strengths of both approaches, such as refining lightweight **semantic segmentation** models with efficient, rule-based post-processing strategies, remains a promising direction. These hybrid methods strike a favorable balance between computational cost and segmentation accuracy, while offering strong potential for generalization across diverse datasets.

To further improve the accuracy of semantic segmentation models, DeepD3 has demonstrated that modifying model architectures can lead to significant performance gains (Fernholz et al., 2024). Its customized network design demonstrates superior performance over the conventional U-Net model, highlighting the effectiveness of architectural optimization.

Improvements in segmentation quality depend not only on model architecture but also on non-architectural factors—such as image preprocessing, training procedures, inference strategies, and post-processing techniques—which play crucial roles. A representative example is the nnU-Net framework, which, although based on the standard U-Net architecture, adapts key non-architectural parameters using rule-based strategies (Isensee et al., 2021). These include image cropping based on object size and optimizing resampling resolutions based on voxel spacing. Such adaptive mechanisms have enabled nnU-Net to achieve leading performance across multiple 3D medical image segmentation tasks, underscoring the importance of these non-architectural aspects in performance optimization.

### The vision and reality of instance segmentation: from ideal goals to feasible path

Semantic segmentation allows classifying pixels as belonging to spines or not. A further level of complexity involves distinguishing individual spines from one to another. To achieve this, instance segmentation can be used to identify distinct spine instances within the ‘spine’ semantic class. For example, the Segmentation Anything Model (**SAM**) (Kirillov et al., 2023), a pre-trained instance segmentation model trained on 11 million 2D natural images, has demonstrated robust generalization capabilities. Subsequently, the Medical SAM Adapter (**Med-SA**) was developed by fine-tuning approximately 2% of the SAM model’s parameters (~13 million), effectively adapting the model to 17 different medical imaging modalities and enabling 3D image segmentation (Wu et al., 2025). These results indicate SAM’s strong potential for cross-domain adaptation and 3D instance segmentation.

However, despite their promise, the practical application of instance segmentation models remains challenging. For instance, training Med-SA requires substantial computational resources (four NVIDIA A100 **GPUs** with 80 GB of memory each). Such hardware is not readily accessible to most researchers or laboratories, especially when compared to the limited resources available on free platforms like Google Colab, which typically offer a single, less powerful GPU (NVIDIA T4) with around 16 GB of memory. Furthermore, **the scale** of existing **3D datasets** for dendritic spine segmentation is significantly smaller than those available in the medical imaging domain. For comparison, the BraTS2021 dataset (Baid et al., 2021) used for training Med-SA includes 1,280 3D samples, whereas spine segmentation datasets are considerably more limited. These challenges in computational resources and data availability constrain the feasibility of adopting instance segmentation models in current research.

As a result, most existing pipelines continue to rely on semantic segmentation, where pixels are labeled as belonging to any spine without distinguishing between individual instances. While effective for detecting the presence of spines, semantic segmentation typically produces a single collective mask, making it difficult to separate adjacent spines, especially when they are densely distributed or intertwined. Given this limitation, rule-based post-processing methods remain essential for refining segmentation results and achieving the necessary isolation of individual spines. Therefore, optimizing **semantic segmentation** pipelines in combination with effective post-processing remains a vital and pragmatic focus for advancing dendritic spine analysis. Achieving **instance segmentation** in the future will depend on continued breakthroughs in models and algorithms, broader access to computational resources, and expansion of datasets. In contrast, achieving **panoptic segmentation** also requires labeling strategies that can clearly show the microenvironment around dendritic spines, such as nearby axons. Membrane labeling techniques can help by providing the information needed enabling precise semantic annotation of each pixel within an image.

### High-quality updated annotation and model adaptation with evolving microscopy technologies

Despite the availability of powerful pre-trained models, annotation and retraining with new datasets is unavoidable. This is primarily due to the continuous advancement in imaging technologies and labelling techniques, which introduce characteristics that differ from those present in the original training sets. As a result, pre-trained models may experience performance degradation when applied to novel datasets. The typical workflow for 3D annotation and model training involves: converting and preprocessing microscopy data, performing semantic annotation with specialized tools, organizing the data into a model-compatible format, and training deep learning models on the annotated dataset (Supplementary Figure S2). Both the quantity and quality of annotated data play critical roles. However, generating precise 3D annotations are time-consuming processes involving meticulous layer-by-layer and pixel-by-pixel inspection.

### 3D annotation platforms

Several bioimage analysis platforms with 3D annotation capabilities have been developed, each offering distinct advantages, as exemplified in Table 1.

**Table 1:** Multi-bioimage analysis platforms provided 3D annotation.

Platform and version	Icy 2.5.2	3D Slicer 5.6.2	Napari 0.4.19	Imaris
License Type	Open-source	Open-source	Open-source	Commercial
Development Stage	Completed	Completed	Napari-nD-annotator under active development	Completed
Operating System	Windows, macOS, Linux	Windows, macOS, Linux	Windows, macOS, Linux	Windows, macOS
Installation Method	Direct installer	Direct installer	Install Napari, then install plugin or additional Python packages	Direct installer
Supported Microscopy Image Formats	Native support for most microscopy formats (e.g., CZI, LIF, TIFF, ND2)	Native support TIFF; Medical imaging formats (e.g., DICOM)	Native support for TIFF; extended microscopy formats (CZI, LIF) require plugins	Native support for most microscopy formats (e.g., CZI, LIF, TIFF, ND2)
Fine 3D Annotation Methods	Manual contour drawing on XY slices	Manual contour drawing with slice interpolation; 3D spherical brush available	Manual contour drawing on XY slices with slice interpolation	Manual contour drawing on XY slices
3D Rendering of Images and Annotations	Supported	Real-time rendering during annotation	Supported	Real-time rendering during annotation
Initial Coarse Annotation Methods	Thresholding, HK Mean clustering	Thresholding	Thresholding	Thresholding
Error Correction Methods	Eraser	3D eraser, 3D scissors	Eraser	3D eraser

3D annotation requires broad support for the import and export of various image formats. For example, the Icy platform provides wild compatibility with microscopy image formats, thereby offering considerable flexibility during annotation (De Chaumont et al., 2012). Efficient initial pre-annotation methods can substantially reduce the manual workload. For instance, the **HK-Mean method** implemented in Icy enables threshold-based preliminary segmentation to expedite this process (Dufour et al., 2008). However, despite the use of automated approaches, substantial manual verification and refinement are still required, rendering the process time-consuming. To accelerate 3D annotations, tools that support efficient interpolation and large-area corrections are highly valuable. A commonly used approach for 3D annotation involves contour-based manual labeling on individual slices. Tools such as Napari and 3D Slicer support automatic interpolation between unannotated slices, thereby improving annotation efficiency (Fedorov et al., 2012; Chiu and Clack, 2022). Notably, 3D Slicer extends the brush tool into a 3D sphere, which enables more efficient volumetric labeling thus speeding up the manual workload.

All platforms provide basic erasing functions, while 3D Slicer additionally supports direct 3D object clipping, enabling precise correction operations. 3D Slicer offers volumetric visualization, facilitating prompt correction, and thereby improving the overall efficiency and accuracy of the annotation process.

In the next years, we may expect that combining semantic segmentation with ruled-based approaches on multi-scale imaging will provide nice information both on neuronal dendritic tree complexity and dendritic spines morphology which are crucial to decipher many neuropsychiatric diseases.

## Glossary

**Related to Advanced Microscopy**
Reconstruction ringing-artifacts: A type of image distortion that appears as spurious ripples or oscillations around sharp edges or high-intensity spots in a reconstructed image. This often occurs due to limitations in the mathematical algorithms (like the Fourier transform) used to process the raw microscopy data, especially when high-frequency information is lost or truncated.
Delaunay triangulation: A computational geometry method for connecting a set of discrete points (or localizations) into a mesh of triangles. Triangles are chosen so that the circumcircle of the triangle should not include any points of neighboring triangles.
Ripley’s function: A spatial statistical method. It is used to assess whether a set of points is clustered, regularly spaced, or randomly distributed. It evaluates the number of neighboring points within a given radius around each point, and compares this to what would be expected under complete spatial randomness. Commonly used in cell biology and neuroscience to analyze the spatial organization of cells, synapses, or molecules.
SODA: SODA stands for Standard Object Distance Analysis. This is a free plugin running on Icy software that use Ripley’s function to analyse spatial distribution of spots or STORM localizations. When used with 2 channel pictures of single molecule Localization microscopy, SODA can indicate whether green molecules are randomly distributed or if they are statistically associated with red molecules. SODA can also be used to check if one protein is associated or not to synapse using a pre or post-synaptic marker (See Lagache et al. Nature Comm 2018 for details or Breton et al. Neurophotonics 2024 for review).
Super-resolution microscopy: A family of light microscopy techniques that circumvent the diffraction limit of light using physical or computational methods to achieve a spatial resolution higher than that of conventional light microscopes (typically <200 nm). This includes SIM, STED, STORM, PALM, SMLM, MINFLUX.
Airyscan: A detector technology for confocal laser scanning microscopy. It replaces the conventional single-point pinhole with an area detector to capture more spatial information. This information is then computationally processed to reconstruct an image with higher resolution and a better signal-to-noise ratio than standard confocal. This technique can be applied to both cultured cells and tissue samples. Resolution typically ranges from 120 to 140 nm. This technique gives higher resolution than confocal but less than “Super-resolution techniques”. Although the resolution is slightly lower compared to SIM, the signal homogeneity is particularly advantageous, as it greatly facilitates image segmentation and quantification.
SIM (Structured Illumination Microscopy): A super-resolution technique. It illuminates the sample with a patterned light (e.g., stripes) causing interference with the sample. By analyzing the " Moiré pattern" created by the interaction of this pattern with the sample's structure, a higher-resolution image is computationally reconstructed from multiple raw images. Resolution typically ranges between 100 and 120 nm. This technique is highly effective in neuronal cell cultures. Its application in tissue samples is possible but more challenging, as artifacts may arise with increasing imaging depth.
STED (Stimulated Emission Depletion): A super-resolution technique. It uses two lasers: an excitation laser to illuminate a spot of fluorescent molecules, synchronized with by a donut-shaped "depletion" laser that deactivates fluorescence in the outer region of the spot, effectively shrinking the point spread function. Resolution typically ranges from 40 to 80 nm. This technique can be applied to both cultured cells and tissue samples. However, imaging thick specimens may require clearing to minimize light scattering and improve acquisition quality.
STORM (Stochastic Optical Reconstruction Microscopy): A single-molecule localization-based super-resolution technique. It uses photo switchable fluorescent probes, stochastically activating only a small, sparse subset of them in each camera frame. By precisely localizing these individual molecules and combining thousands of such frames, a high-resolution image is reconstructed. Resolution typically ranges from 10 to 50 nm. This technique can be applied to both cultured cells and tissue samples. However, imaging thick specimens is challenging (due to blinking buffer penetration for STORM, or oligos penetrations for PAINT) and light scattering.
MINFLUX (MINimal emission FLUXes): A super-resolution technique that combines the principles of STED and single-molecule localization. It uses a donut-shaped excitation beam (dark in the center and bright around it) to probe fluorescence at different positions. By fitting photon count distributions obtained from multiple targeted excitation positions surrounding the fluorophore, the system triangulates the molecule’s location with high precision and low photobleaching due to reduced excitation intensity. It can achieve 1-3 nm precision and is fast enough to track molecules live. Most published applications concerns cell culture up to now.
SNR (Signal-to-Noise Ratio): A measure of signal quality. It compares the level of a desired signal to the level of background noise. A high SNR indicates a clean and reliable measurement.
PSF (Point-Spread Function): This is a mathematical function that describes how the image of a single point is distorted by a microscopy system. For example, a fluorescent spherical bead with a physical diameter of 100 nm appears larger than 200 nm in fluorescence microscopy. The three-dimensional image of such a bead—commonly referred to as the point spread function (PSF)—reveals that the distortion is typically anisotropic in conventional microscopy, with greater deformation along the Z-axis than in the X and Y directions.
**Related to Image Segmentation & Deep Learning Topic**
HK-Mean: This segmentation method applies N-class thresholding based on K-Means clustering of the image histogram. It then performs object extraction in a bottom-up manner, guided by user-defined minimum and maximum object size constraints. Conventional thresholding typically employs two classes, with black representing the background and white denoting the object of interest. By using HK-Means clustering, for example, it becomes possible to perform thresholding with four classes: white, black, and two intermediate grey levels.
FCN (Fully Convolutional Network): A type of neural network model where all layers perform convolutions. Unlike traditional networks that have fixed-size outputs, FCNs can process input images of any size and produce an output map of a corresponding size (e.g., a segmentation mask). This makes them ideal for pixel-level tasks like identifying all cells in an image.
U-Net: A widely-used neural network model for biomedical image segmentation, named for its U-shaped architecture. The U shape is composed of 2 parts: the stem (the U center that extract interesting characteristics) and the decoders that extend resolution by up-sampling. Its design cleverly combines an understanding of the overall image context with the preservation of fine details, allowing it to achieve highly precise segmentation even with limited training data. This makes it particularly effective in fields like medicine and biology where large annotated datasets are often rare.
Dual-decoder network: An efficient neural network architecture designed to perform two related output tasks simultaneously from a single image. It operates in two steps: first, a shared *Encoder* analyzes the image to extract key feature information, performing an "understanding" role. Then, two separate *Decoders* use this shared information to construct different output maps. For instance, one decoder can generate the precise boundaries of cells (*a boundary map*), while the second decoder simultaneously generates a map identifying different cell types (*a class map*).
Panoptic segmentation: An image segmentation goal. It doesn't just distinguish "cell" from "background" (semantic segmentation), but also identifies each individual cell instance. The final result: every pixel in the image is labeled as either background or as belonging to a specific instance, like "cell 1", "cell 2", etc. This is crucial for accurate counting and single-cell analysis.
SAM (Segmentation Anything Model): A foundation model for image segmentation. It was pre-trained on a massive general-purpose dataset containing 11 million images and over 1 billion segmentation masks. This extensive training equips it with powerful "zero-shot" segmentation capabilities, allowing it to segment virtually any unseen object in an image, often prompted by minimal user input like a single click or a box.
Med-SA (Medical SAM Adapter): An efficient method for adapting the general-purpose SAM for medical image analysis. It enables the model to process various types of 3D medical data—a task the original 2D SAM cannot perform.
Med-SA fine-tuning: "Fine-tuning" means taking a smart model (here, Med-SA) that has already been trained on a vast amount of general medical images, and then giving it specialized training on our own smaller, specific dataset (e.g., our images of a particular neuron type). This allows the model to quickly adapt and become an expert on our unique analysis task without starting from scratch.
GPU memory: This refers to the dedicated, high-speed memory on a Graphics Processing Unit (GPU). In deep learning, GPUs perform massive parallel computations. GPU memory acts as the GPU's "workbench"; its size determines the size and complexity of the images and neural network models that can be processed at once. Large GPU memory is critical for handling high-resolution 3D images or large models.
Dataset scale: Refers to the amount of data used for training and testing a model. It can be measured in various ways, such as the total number of images, the total number of annotated objects, or the total data storage size (e.g., Gigabytes, Terabytes). The scale of the dataset directly impacts the complexity of the computational methods that can be applied and the robustness of the resulting model.
