# Supplementary material for: Super-resolution microscopy and deep learning methods: what can they bring to neuroscience: from neuron to 3D spine segmentation
Source: Front Neuroinform. 2025 Sep 29;19:1630133. doi: 10.3389/fninf.2025.1630133 (PMC12515862; doi:10.3389/fninf.2025.1630133)
Supplement: Supplementary file 1 [file Data_Sheet_1.pdf]

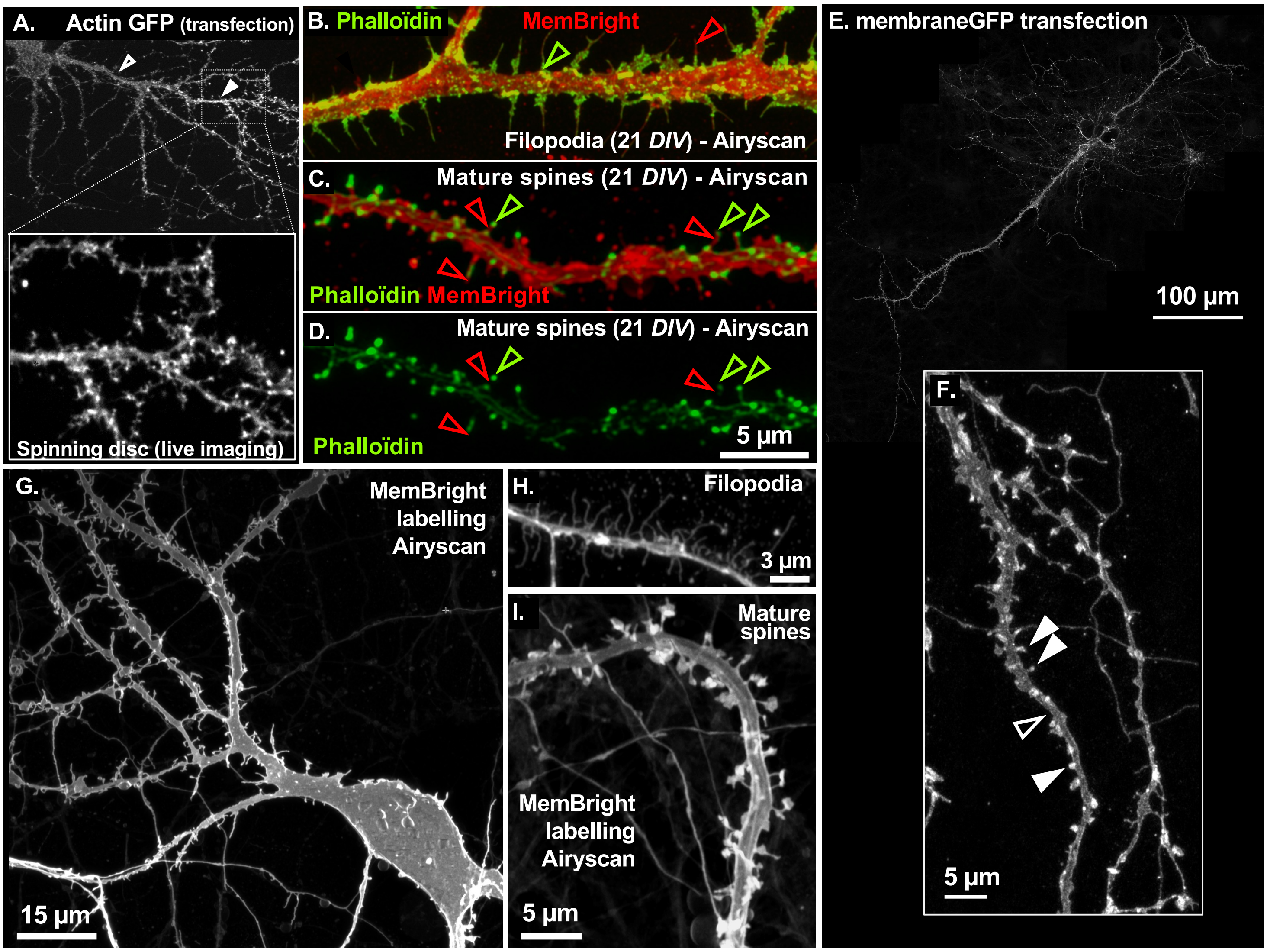

Figure S1 : Strategies for imaging and segmenting neurons.
A. Live hippocampal neurons were transfected with actin GFP and imaged lived using spinning-disc microscopy. Actin is labeling neuronal dendrite with variable intensity depending on the dendrite segment (arrows). Accumulation of actin is visible in dendritic spine heads decorating the dendrite. **B-C-D.** Live neurons were labeled with MemBright probes (in red), then fixed and labelled with phalloïdin (in green). Filopodia are visible in B, while mushroom spines with bulbous heads filled with actin (green arrow) are present in C and D. MemBright labeling allows the detection of dendritic shaft and filopodia or spine neck that are faintly visible with phalloidin (red arrows in C and D). **E-F.** hippocampal neuron transfected with membranous GFP showing higher signal in dendritic spines (white arrows) than in dendritic shaft (black arrow). **G-H-I**. live hippocampal neuron labeled with MemBright probe and imaged using Airyscan microscopy. MemBright reveals both cell body, dendrite (G) and membrane protrusions (H and I). This quite homogeneous signal allowing accurate segmentation. Tiny spine neck close to bulbous spine head can be seen on mushroom spines in I.

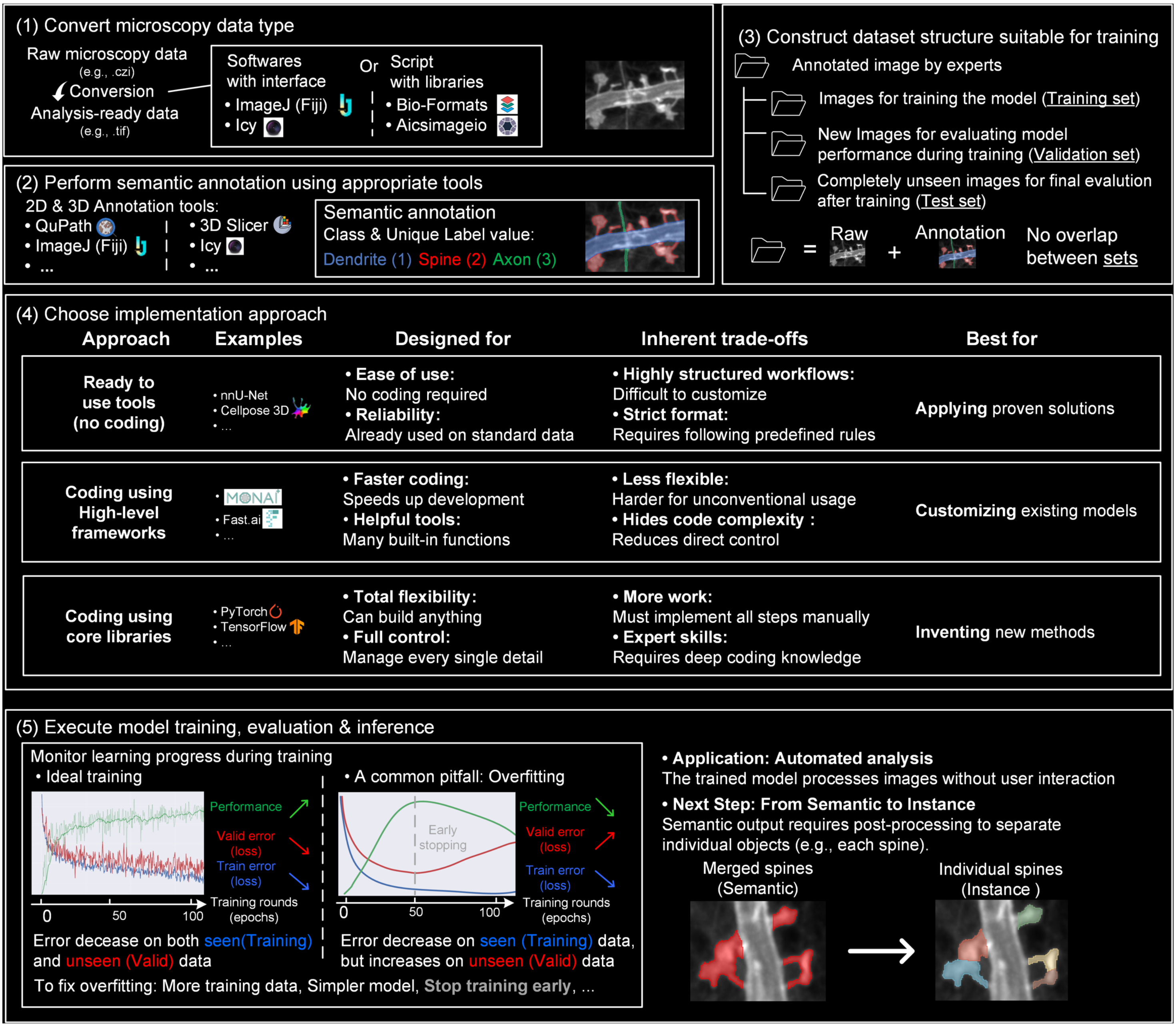

Figure S2 : Deep Learning (DL) pipeline: From Acquisition & Annotation to Training & Inference
The process begins with data preparation, which includes (1) converting raw microscopy data into an analysis-ready format and (2) performing semantic annotation using specialized 2D or 3D tools. The annotated data is then partitioned into distinct (3) training, validation, and test sets for robust model development and evaluation. A key strategic step involves (4) choosing an implementation approach, weighing the trade-offs between ready-to-use tools for rapid application, high-level frameworks for customization, and core libraries for novel development. The final stage (5) covers both model training and application. During training, progress is monitored to avoid common pitfalls like overfitting. Once trained, the model is used for automated inference, but its semantic output often requires a post-processing step to separate individual instances (e.g., each spine) from a single "all spines" mask.
